# Supplementary material for: Comparative evaluation of five rapid PCR platforms for respiratory virus detection
Source: PLoS One. 2025 Dec 11;20(12):e0338716. doi: 10.1371/journal.pone.0338716 (PMC12697972; doi:10.1371/journal.pone.0338716)
Supplement: Attachment — Submitted filename: rebuttal letter.docx [file pone.0338716.s001.docx]

Dear Editor and Reviewer,

Thank you for the careful and constructive review of our manuscript (Title: Comparative Evaluation of Five Rapid PCR Platforms for Respiratory Virus Detection, Manuscript ID: PONE-D-25-41222). We appreciate the reviewers’ time and comments. We have prepared a point-by-point response (attached) and made the following key revisions to the manuscript:

- Emphasis on negative samples: We clarified and strengthened the presentation regarding the absence of negative samples and discussed its implications throughout the Methods and Results sections.
- Statistical analysis: We added appropriate statistical analyses to support our findings (see Methods).
- Study design and limitations: We expanded the Discussion to explicitly acknowledge limitations of the study design, including potential biases and constraints that affect interpretation and generalizability.
- Costs and user operability: The Results section now places greater emphasis on cost considerations and practical aspects of user operability, with additional text quantifying or qualifying these factors.
- Additionally, when preparing the raw data file, we cross-checked again all data and decided to represent the data slightly differently for easier representation in combination with the raw data. This had marginal effect on the total number of negative samples tested and no effect on our findings. To reflect this update, we have corrected Table1.

All reviewer comments have been addressed point-by-point in this document. Where we did not follow a suggested change, we provide a brief justification.

We believe these revisions improve the clarity and robustness of the manuscript and welcome further feedback.

Sincerely,

Dr. P. Smit

Dr. D.S.Y. Ong

**Point by point rebuttal below**

When submitting your revision, we need you to address these additional requirements.

Please ensure that your manuscript meets PLOS ONE's style requirements, including those for file naming. The PLOS ONE style templates can be found at

<https://journals.plos.org/plosone/s/file?id=wjVg/PLOSOne_formatting_sample_main_body.pdf> and

[https://journals.plos.org/plosone/s/file?id=ba62/PLOSOne_formatting_sample_title_authors_affiliations.pdf](https://track.editorialmanager.com/CL0/https://journals.plos.org/plosone/s/file)

**RESPONSE**

We have checked the PLOS ONE’s style requirements and adopted accordingly.

2. We note that your Data Availability Statement is currently as follows: “All relevant data are within the manuscript and its Supporting Information files”

Please confirm at this time whether or not your submission contains all raw data required to replicate the results of your study. Authors must share the “minimal data set” for their submission. PLOS defines the minimal data set to consist of the data required to replicate all study findings reported in the article, as well as related metadata and methods (<https://journals.plos.org/plosone/s/data-availability#loc-minimal-data-set-definition>).

For example, authors should submit the following data:

- The values behind the means, standard deviations and other measures reported;

- The values used to build graphs;

- The points extracted from images for analysis.

Authors do not need to submit their entire data set if only a portion of the data was used in the reported study.

If your submission does not contain these data, please either upload them as Supporting Information files or deposit them to a stable, public repository and provide us with the relevant URLs, DOIs, or accession numbers. For a list of recommended repositories, please see <https://journals.plos.org/plosone/s/recommended-repositories>.

If there are ethical or legal restrictions on sharing a de-identified data set, please explain them in detail (e.g., data contain potentially sensitive information, data are owned by a third-party organization, etc.) and who has imposed them (e.g., an ethics committee). Please also provide contact information for a data access committee, ethics committee, or other institutional body to which data requests may be sent. If data are owned by a third party, please indicate how others may request data access.

**RESPONSE**

We appreciate the effort of Plos One in making underlying data more accessible and transparent. In that regard, we have added the raw data in a supplementary table 1.

3. We note that you have included the phrase “data not shown” in your manuscript. Unfortunately, this does not meet our data sharing requirements. PLOS does not permit references to inaccessible data. We require that authors provide all relevant data within the paper, Supporting Information files, or in an acceptable, public repository. Please add a citation to support this phrase or upload the data that corresponds with these findings to a stable repository (such as Figshare or Dryad) and provide and URLs, DOIs, or accession numbers that may be used to access these data. Or, if the data are not a core part of the research being presented in your study, we ask that you remove the phrase that refers to these data.

**RESPONSE**

We deleted the sentence that referred to the data not shown comment in the discussion as requested, because it did not belong to the core part of the research.

4. If the reviewer comments include a recommendation to cite specific previously published works, please review and evaluate these publications to determine whether they are relevant and should be cited. There is no requirement to cite these works unless the editor has indicated otherwise.

**RESPONSE**

We thank the editor for this remark. The reviewer has not given a recommendation to cite specific published work.

5. Please review your reference list to ensure that it is complete and correct. If you have cited papers that have been retracted, please include the rationale for doing so in the manuscript text, or remove these references and replace them with relevant current references. Any changes to the reference list should be mentioned in the rebuttal letter that accompanies your revised manuscript. If you need to cite a retracted article, indicate the article’s retracted status in the References list and also include a citation and full reference for the retraction notice.

**RESPONSE**

Thank you, we have checked the reference list and we do not have retracted cited papers.

Additional Editor Comments:

The manuscript presents an informative comparative evaluation of diagnostic performance across various platforms. However, inclusion of complete negative sample data is essential to enable a comprehensive specificity analysis and strengthen the overall conclusions.

**RESPONSE**

We agree with the editor that a full prospective study that would also consecutively include all negative samples would have been preferred. However, with an expected prevalence for each of the different viruses, this would have dramatically increased the sample size to end up with a sufficient number of positive samples or it would have led to lower number of positive samples, thereby limiting the accurateness of the sensitivity assessment. However, to emphasize this limitation, we included a sentence in the discussion:

*“A separate group of samples negative for all viral targets was not included in this study. However, for each pathogen, negative results were inherently represented by samples positive for other viral targets, ensuring that each assay’s specificity could still be evaluated within the tested cohort. Nevertheless, the absence of a distinct all-negative control group may limit a full assessment of assay performance in populations with low pathogen prevalence. “*

The primary objective—to assess diagnostic accuracy, usability, and operational costs—has been stated but is not clearly reflected in the Results or Discussion sections. The current focus remains largely on positive samples; therefore, inclusion of negative sample testing and elaboration on usability and cost parameters would provide a more balanced interpretation.

**RESPONSE**

We agree with the editor that our comment on costs and usability was very short. We have added the following to the result section:

“According to laboratory technicians’ experience during the evaluation, the Galaxy Lite platform was less user-friendly than the other systems, requiring additional manual steps and being the only assay that could not be stored at room temperature. In the Dutch laboratory setting, all newly introduced platforms were less expensive per test than the GeneXpert platform, although such price differences may vary across countries and healthcare systems.”

For samples that tested positive on one platform but were undetected on others, it is recommended to repeat testing at least once to confirm negative results, thereby improving data reliability.

**RESPONSE**

We agree with the reviewer from a scientific point of view that this would have been of added value. In this study we wanted to mimick as closely as possible to the clinical use case of these rapid diagnostic tests and decided to do this prospectively in a routine hospital laboratory setting. In almost all clinical settings, a test result would not be repeated if negative. Therefore, we kept the result as they were. However, in case a test failed, we repeated the sample. This would also represent real-world clinical practice as otherwise no test result could be reported to the clinicians.

We have added a note on this in the method section:

“To represent most clinical settings, invalid test results were repeated, but discrepant test results in comparison to other platforms were not repeated.”

Additionally, the rationale and methodology for sample size calculation should be included to enhance transparency and reproducibility. In the Results section, a more detailed statistical analysis is advised to improve clarity and interpretability of findings.

The authors are also requested to carefully address all reviewer comments and revise the manuscript in accordance with the journal’s publication guidelines.

**RESPONSE**
We value the suggestions from the editor and added a sample size calculation.

“Sample size calculation was performed with the aim to be able to detect at least a 15% difference in agreement per viral target with 80% power and an α of 0.05 (McNemar), which showed that 53 samples were required per viral target.”

Regarding the question of the editor to add statistical analyses, we kindly disagree that adding statistics to this relatively plain and straightforward manuscript would be of any added value. In our opinion we do not make a strong statement that needs to be supported by any statistics and therefore see no added value.

Reviewer's Responses to Questions

**Comments to the Author**
Reviewer #1: This study offers valuable comparative data on emerging rapid PCR platforms for respiratory virus detection. The manuscript is well-organized and clearly written, but several points need clarification.

The study aims to compare diagnostic performance against GeneXpert but only includes positive samples, which limits the assessment of specificity and predictive values. The authors should clearly state in the Methods section that only GeneXpert-positive samples were included and discuss this limitation in the Discussion. Additionally, the rationale for the number of samples per virus should be clearly explained.

**RESPONSE**

We thank the reviewer for the kind words and the suggestion to describe more clearly this limitation in the method and discussion sections. The discussion section was already adjusted based on the editors comments , see comment above. And below:

*“A separate group of samples negative for all viral targets was not included in this study. However, for each pathogen, negative results were inherently represented by samples positive for other viral targets, ensuring that each assay’s specificity could still be evaluated within the tested cohort. Nevertheless, the absence of a distinct all-negative control group may limit a full assessment of assay performance in populations with low pathogen prevalence. “*

Based on this comment we adusted the method section. It now reads:

“Positive samples according to GeneXpert were enrolled consecutively until the target number per virus was reached. No separate group of negative samples was included.”

The description of the statistical analysis is brief and should specify how agreement percentages were calculated, such as whether based on simple proportion, Cohen’s kappa, or diagnostic accuracy metrics like sensitivity and specificity. It should also clarify how invalid or failed runs were handled in the analysis, whether they were excluded or counted as test failures, especially since test failures were only reported for the Galaxy Lite platform (27.2%), while the other systems nearly always produced valid results. This clarification would improve methodological transparency.

**RESPONSE**
Thank you for these suggestions, we have included a statement regarding the invalid test results based on editors suggestion (see above) and invalid test results are also shown in table 1.

The method section now reads:

“Key performance metrics such as concordance and percentage agreement ratios, including qualitative assessments of user experiences and operational reliability, were assessed for each platform”

The results are generally appropriate for the study’s comparative aims and show clear differences in performance between platforms. However, because only GeneXpert-positive samples were included and no true negative group was tested, the study cannot evaluate specificity or predictive values. While reporting agreement percentages is suitable for comparative evaluation, this limitation should be explicitly acknowledged in the Discussion to prevent overinterpreting the diagnostic accuracy. Additionally, the small sample size for some subgroups and the exclusion of Galaxy Lite due to high failure rates should also be acknowledged.
 The findings are acceptable and informative for preliminary evaluation but should be interpreted cautiously in the absence of a full positive-negative dataset.

**RESPONSE**

Thank you for this suggestion which fits with the suggestions of the editor. We have made this limitation more explicit in the discussion. It now reads:

*“Limitations include the absence of blinding, predefined group sizes, and limited sample numbers for certain platforms (e.g. SD Biosensor with two different kits) or subgroups of respiratory viruses. A separate group of samples negative for all viral targets was not included in this study. However, for each pathogen, negative results were inherently represented by samples positive for other viral targets, ensuring that each assay’s specificity could still be evaluated within the tested cohort. Nevertheless, the absence of a distinct all-negative control group may limit a full assessment of assay performance in populations with low pathogen prevalence. “*
